# On Perityphlitis

**Published:** 1880-12

**Authors:** Henry B. Sands

**Affiliations:** Professor of the Practice of Surgery in the College of Physicians and Surgeons, New York; Attending Surgeon to the New York and Roosevelt Hospitals


### Selections.

ON PERITYPHLITIS. By HENRY B. SANDS, M.D., Professor of the Practice of Surgery in the College of Physicians and Surgeons, New York; Attending Surgeon to the New York and Roosevelt Hospitals.

Since the year 1867, when Dr. Willard Parker happily advocated and adopted the plan of treating perityphlitic abscesses by early incision, the procedure has received considerable attention from American surgeons, and the practice recommended by Dr. Parker has been fully indorsed by all who have published the results of their experience. That the disease, however, although by no means uncommon, is frequently overlooked or misunderstood, seems quite certain; and what is known about it has not yet been embodied in the standard surgical text-books, but remains scattered among the pages of our current medical literature. Further discussion of the subject is therefore desirable, in order that the disease may be readily recognized and submitted to appropriate surgical treatment; and in order that those cases in which operative interference is necessary may be, if possible, distinguished from others, not infrequent, which tend to spontaneous recovery, or which, at least, are likely to terminate favorably without the aid of the knife. My object in the present communication is briefly to present to the Society the results of my own observation of this affection, in the hope of eliciting profitable discussion, and of obtaining such additional information derived from the personal experience of my colleagues as shall throw light upon several obscure and unsettled points connected with its pathology and treatment. My remarks will refer only to those cases of inflammation in the neighborhood of the cæcum, in which the disease is circumscribed; as those in which general peritonitis rapidly follows a perforation of the cæcum or the vermiform appendix, belong to a separate category, and terminating fatally, are as yet beyond the reach of art, and possess therefore a pathological rather than a surgical interest.

Thus defined, twenty-six cases of perityphlitis have fallen under my notice. Of these, twenty-two were observed in males and four in females, thus confirming the fact already established concerning the comparative rarity of this disease in the female sex. Of the entire number, only one occurred in hospital practice. The youngest patient was nine years of age, and the oldest fifty-four. Of the rest, ten were between ten and twenty, seven between twenty and thirty, two between thirty and forty, and five between forty and fifty.

As is well known, various causes have been assigned by systematic writers for the occurrence of the inflammatory affection under consideration, and a pathological classification of the cases I have seen would doubtless be preferable to any other, provided it could be accurately made. But as in all, except three of them, the disease terminated in recovery, the precise pathological condition could rarely be determined; and therefore I have thought it more profitable to separate the cases into four divisions, as follows:

1. Cases terminating in resolution, without evidence of suppuration.
2. Cases of abscess, terminating in spontaneous recovery.
3. Cases of abscess, treated by operation.
4. Cases of abscess, unopened, and ending fatally.

The first group comprises ten cases, in all of which the disease disappeared without showing any signs of suppuration. Inasmuch as many persons entertain the erroneous notion that perityphlitis, when once established, must necessarily terminate in the formation of abscess, it will be important to dwell on these cases long enough to show that their diagnosis was carefully and fairly made out. In all, the following symptoms were present, namely: abdominal pain and tenderness, usually occurring suddenly, sometimes limited to and always most marked in the region of the cæcum; fever, indicated by an acceleration of pulse and a rise of temperature; and the presence of an indurated swelling, distinguishable either by palpation in the right iliac fossa or by digital exploration of the rectum. In most of the cases, the onset of the disease was severe, being characterized by nausea and vomiting, and by acute pain and tenderness in the cæcal region. Sometimes the pain in the abdomen was diffused in the beginning, and became localized only on the second or third day. The temperature was elevated in every case, ranging from 100° to 104° F., in one case reaching 105°. The most striking feature of the disease, namely, the presence of a circumscribed induration in the right iliac fossa, was observed in nine out of the ten cases belonging to this group; while, in the remaining case, the inflammatory swelling could be distinctly felt through the rectum, the finger detecting a firm elastic mass, tender to the touch, and evidently developed from the region of the caput coli. When present in the iliac fossa, the tumor seemed deep-seated, and was invariably situated above and within a short distance of Poupart's ligament. Its external margin often advanced to a point within an inch of the anterior spinous process of the ilium, while, internally, it seldom reached the median line, in one instance, however, passing two inches beyond it. Its upper limit rarely extended above the cæcum or adjacent part of the ascending colon. In all instances the tumor was deep-seated, immovable, and tender on pressure; and in several the abdominal wall covering the tumor anteriorly was so exceedingly painful to the touch as to render a thorough examination quite difficult. The induration was usually discovered within forty-eight hours after the commencement of the disease, and generally began to subside soon after the abatement of the other symptoms.

It is worthy of notice, however, that in almost all cases the subsidence of the tumor was gradual, and that in one instance nearly five months elapsed before the disappearance was complete. This fact, coupled with the circumstance that care was always taken to secure a thorough action of the bowels by the administration of purgatives, clearly indicates the inflammatory nature of the tumefaction, and forbids the supposition of the latter being directly due to fæcal impaction in the cæcum. In one exceedingly chronic case, in which the disease lasted for several months, an accumulation of hardened fæces in the cæcum and colon seemed evidently the origin of the inflammatory mischief, which continued, however, long after the exciting cause had been removed. So far as the early symptoms are concerned, these cases could not be distinguished from those that were destined to go on to suppuration. The abdominal pain, the fever, and the gastric disturbance were quite as severe, and the indurated mass occupying the iliac fossa presented the same characters. I have therefore learned to decline, in the beginning of the disease, to express a definite opinion as to the probable ultimate result, since time alone can decide whether the inflammatory process shall be arrested, or advance to the stage of suppuration. Fortunately, the doubt is often solved at an early period, for I ascertain from my notes that in five out of the ten cases resolution occurred as early as the fifth, and not later than the eighth day; while in only one case was it delayed beyond the fourteenth day. The favorable termination was indicated by a decided fall in the pulse and temperature, the latter sometimes dropping below the normal standard, and by a marked diminution of abdominal tenderness, pain and distension. These signs of improvement generally coincide with a decrease of the local tumefaction, traces of which, however, as has been mentioned, often remained long after the establishment of convalescence. In a few instances the tumor subsided so rapidly as to suggest that an abscess had ruptured, although a careful inspection of the urine and fæces failed to detect admixture of either blood or pus.

Regarding the pathology of the cases now referred to, nothing very definite can be stated. In one case, impaction of fæces in the cæcum seemed a probable cause; in another, the disease came on after unusual muscular exertion; while in a third, it may have been due to some intestinal lesion accompanying continued fever. In the remaining seven cases, no adequate cause could be discovered. In all, however, the symptoms pointed to plastic inflammation of the connective tissue adjacent to the cæcum as the proximate cause of the characteristic induration which, with a single exception, was found in the iliac fossa. In this exceptional case, in which the inflammatory swelling could be felt only through the rectum, it may be conjectured that the irritation proceeded directly from the vermiform appendix which, instead of being curled up behind the cæcum, lay at, or below the level of the brim of the pelvis. What proportion of cases were connected with the lodgment of fæcal concretions or foreign bodies in the vermiform appendix cannot, of course, be determined; but it should be remembered that this morbid condition is the most frequent cause of perityphlitis when the disease culminates in abscess, and that in many of the latter class of cases, there is obtained a history of one or more preceding attacks of a similar character, terminating by resolution. The invariable occurrence of such disease on the right side can be explained only by reference to the cæcum or its appendix as the starting point; and pathological anatomy has demonstrated that lesions of the appendix are relatively more frequent, as well as more dangerous than those of the cæcum itself.

The treatment of the cases which terminated in resolution consisted mainly in local depletion, the application of fomentations or poultices to the abdomen, and the internal administration of opium. Eight or ten leeches were usually applied over the cæcal region, and sometimes the leeching was repeated. The effect was often markedly beneficial. Opium or morphia was given in moderate doses, sufficient to allay pain, but never so as to induce narcotism. Castor oil, fluid extract of senna, or calomel was administered in nearly all cases early in the disease, and I would remark that this practice appears to be judicious for two reasons: first, to afford aid in diagnosis, by excluding fæcal impaction in the cæcum as a possible cause of the symptoms; and secondly, because any accumulation of fæces in the intestines increases the patient's suffering, and is doubtless liable to aggravate the existing inflammatory mischief. The propriety of using cathartics in this affection has been often doubted, and we are taught by some that here, as in the case of general peritonitis, they should be absolutely withheld. But the analogy between the two diseases is not very close, and experience has shown that the fear which has been expressed concerning the danger of cathartics in perityphlitis is unreasonable. Nothing can be gained, however, by their repeated administration; a single purgative, given in the beginning of the disease, will generally suffice to empty the bowels, while subsequent accumulation can be obviated by careful restriction of the diet, and by the selection of light, nutritious articles of food. The treatment thus briefly outlined has been occasionally supplemented by the use of blisters and mercurial applications, whenever the absorption of the inflammatory products appeared to be unduly retarded.

The recognition of the class of cases first described is important, on account of the erroneous opinion widely entertained, that perityphlitis, when once established, must necessarily proceed to suppuration. While this may, perhaps, be true when the disease is due to perforation of the intestine, with the consequent escape of its contents into the surrounding tissues, it is quite exceptional in those cases which owe their origin to the mere presence of hard and indigestible substances in the cæcum or its appendix, to the extension of catarrhal processes in the cæcum through the intestinal coats, to the effects of injury, or to causes which, with our present lack of knowledge, we are unable to define, but yet cannot refuse to acknowledge. Such cases, tending to recovery without suppuration are, as my own experience proves, by no means rare, and their relative frequency is doubtless greater than my figures would seem to indicate, inasmuch as the surgeon is apt to witness the severer, rather than the milder examples of the disease. Since many of them terminate favorably before the end of the first week, the question of the propriety of surgical intervention will often not arise, but when resolution is deferred to the tenth, twelfth or fourteenth day, or even later, it will often require nice judgment to decide whether to operate or not, especially as the surgeon is taught, in doubtful cases, to resort to the knife, without waiting to detect fluctuation, and not to delay the incision much beyond the first week of the disease. It must be evident, however, that the question of operation is one that cannot be settled by time alone, and that all the circumstances of the case should be carefully considered before resorting to a procedure which may be needless and possibly hazardous. This subject naturally leads to the study of the cases forming the second group, namely, those of abscess terminating in spontaneous recovery.

I have already remarked that in several cases included in the first series, the subsidence of the local swelling took place so rapidly that it was hard to believe an abscess had not ruptured and emptied its contents into some neighboring hollow viscus. But as an examination of the urine and fæces afforded no evidence in support of such a view, the question was necessarily left in doubt. In three instances, however, my notes furnish conclusive proof that such an event may happen and that it may be followed by complete and speedy recovery. Many years ago I attended a lady afflicted with an abscess in the cæcal region, which, at the end of the second week, was accompanied with alarming symptoms of general peritonitis. Suddenly a large quantity of pus was discharged at stool, and the patient, whose condition had hitherto seemed desperate, became immediately convalescent. In the year 1876, I saw, in consultation with Dr. Pierson, of Orange, a girl nine years of age, who had suffered during the previous eight days with the usual symptoms of perityphlitis. At the time of my visit I discovered a non-fluctuating, tender swelling in the iliac fossa. The severity of the general symptoms, as well as the abdominal pain and distension, led me to suspect the existence of an abscess, which I succeeded in demonstrating by employing the hypodermic syringe as an aspirator and withdrawing a small quantity of pus. I paid a second visit to the patient on the following day, intending to open the abscess in the usual manner, but found, on my arrival, that the tumor had almost entirely disappeared. I was informed that during the preceding night the child had grown rapidly worse and complained of intense pain in the region of the bladder. Soon afterward she voided a very large quantity of urine, and then at once became quiet and free from pain. Unluckily, the nurse threw away the urine without examining it, but we inferred that the abscess had ruptured into the bladder. In any case, it must have discharged its contents at the time stated, for on the following morning the tumor could hardly be felt, the pulse and temperature had declined to the natural standard, abdominal pain and distention had disappeared, and the patient had evidently passed the crisis. The third case I saw last year in consultation with Dr. M. R. Vedder. The patient was a girl thirteen years old, who had been attacked with perityphlitis a week previously.

I found the usual tumor well marked in the iliac fossa, and also unusually prominent in the rectum. A purgative was prescribed which acted freely, and on the ninth day of the disease the swelling almost disappeared, and the fæcal evacuations, on being examined, were seen to contain a notable quantity of blood and pus. In this case the abscess probably broke into the rectum, and, as in the two other instances just related, the patient speedily regained her health.

These three cases prove that even when a perityphlitic abscess is left to itself, its course is not always unfavorable, but that its contents may be discharged into the intestine or possibly into the bladder, without any serious consequences. Indeed, it would appear that this mode of termination is adapted to secure the best possible result, for convalescence begins as soon as the abscess has ruptured, whereas, after an external opening has been established either naturally or by incision, the suppuration is always more or less protracted and the cure correspondingly retarded. Unfortunately we are unable, in any given case, to predict this result, which, moreover, is so exceptional in its occurrence that it does not invalidate the rule of affording vent to the matter by an external incision, so soon as the diagnosis of abscess is reasonably sure.

Cases of abscess treated by operation. This group contains eleven cases, the study of which reveals many interesting facts. The characteristic tumor in the iliac region was present in all the cases, but in only two was any swelling discoverable on digital exploration of the rectum. In four instances there was fluctuation. In one of these the date of operation was not recorded, but it was late in the disease, and the integument was extensively undermined, nevertheless the patient recovered. In the three remaining cases the abscess was opened on the fifteenth day, the seventeenth day, and at the end of the ninth week, respectively. The last named case, in which the operation was so long delayed, terminated fatally by septicæmia. At an earlier stage of the disease, and before fluctuation was evident, I proposed an exploratory incision, but the patient refused to submit to it. When, at last, the abscess pointed over the middle of the crest of the ilium, it had already burrowed extensively and acquired extraordinary dimensions. After being opened, it continued to discharge very copiously, and, in spite of the employment of antiseptic injections, septicæmia occurred and carried off the patient. The case is instructive as illustrating the danger of delay, for it is the only one out of the entire number embraced in the present group in which death followed the operation. Had the abscess been opened at an earlier period, a fatal termination would probably have been averted.

In the remaining seven cases that were treated by incision, fluctuation was absent, and there does not appear to have been any one symptom indicating that suppuration had taken place. Yet in only one instance did the knife fail to penetrate an abscess, a circumstance which shows that the diagnosis can be made out with tolerable certainty, even when demonstrative evidence is wanting. Rigor, sweating, high temperature, acceleration of pulse, abdominal pain and tympanites, and an increasing extent combined with diminishing firmness of the abdominal tumor, are the chief signs which indicate the formation of pus. But none of these signs is invariably present, and it would be a difficult matter to say which one of them is the most important. But although in the early stage of the disease it may be impossible to discriminate between the cases that are going to terminate by resolution and those that are to end in suppuration, the latter may usually be distinguished toward the close of the second week by the generally unfavorable condition of the patient, who seems to be growing worse instead of better; whereas, when resolution is about to take place, the later course of the disease is comparatively mild and favorable. In one remarkable case already mentioned, wherein the affection continued for many months and ended without suppuration, the combination of symptoms was never such as to demand surgical interference, although on two occasions I was nearly persuaded to undertake an exploratory operation.

The seven cases now under consideration were treated by incision as follows: one on the ninth day, two on the twelfth day, one on the thirteenth day, and two on the twenty-first day. In all cases except the first, the abscess was found and opened. In the one in which the incision was made on the ninth day, no abscess could be discovered, although the knife was carried through the fascia transversalis, and the hypodermic needle thrust in various directions in the hope of finding pus. After the operation the patient grew worse, and his life was despaired of, when, eleven days later, an abscess broke and discharged its contents through the wound. Perhaps, in this instance, the operation was serviceable by dividing dense structures which might have offered resistance to the progress of matter toward the external surface, but it would, of course, have been more gratifying if an abscess had been reached at once. Usually a perityphlitic abscess remains of moderate size until about the end of the second week, and by deferring an operation until it is ripe, we shall find the deeper textures consolidated and agglutinated by plastic lymph, and, therefore, less liable to be infiltrated by the fetid discharges which, after incision, often cause more or less sloughing of the margins of the wound. On the other hand, the danger that the abscess, if unrelieved, may rupture into the peritoneal sac must not be forgotten. Through the kindness of Dr. Wiener I have the opportunity of showing to the Society a specimen illustrating this unfortunate accident. On Tuesday last, Dr. Wiener was called to see a gentleman who had been ill for six days with perityphlitis. The characteristic tumor was present in the iliac fossa, and the case being regarded as one of abscess, arrangements were made to open the latter on the following day. During the night, however, in consequence, it is supposed, of some incautious movement made by the patient, rupture into the peritoneum took place, and death ensued ten days afterward. The bursting of the abscess was indicated by a disappearance of the tumor and by collapse, followed by the usual symptoms of acute peritonitis. I believe such an event as this is very rare, but the possibility of its occurrence must make us watchful and anxious until the crisis is past. Everything depends on an exact diagnosis and on an early recognition and treatment of existing abscess, and I would suggest a more frequent employment of the aspirator as affording the most reliable test at our command for purposes of diagnosis. The smallest needle will suffice, and can be repeatedly inserted, if necessary, without doing harm. By means of this instrument the situation, as well as the presence of an abscess, can be determined, and it is well known that these collections of matter do not always occupy the same locality.

In every case that has fallen under my observation, the operation has been performed essentially in the manner recommended by Dr. Parker. An incision several inches in length, and usually parallel with Poupart's ligament, was made over the most prominent part of the tumor, through the skin and sub-cutaneous fat. Subsequently the deeper layers were divided until the abscess was reached, or until the fascia transversalis came into view. If fluctuation then became evident, the abscess was immediately opened, otherwise the fascia was penetrated in various directions by means of a hypodermic syringe until the seat of the abscess was discovered, when the operation was completed by entering a narrow bistoury alongside of the needle. I have no doubt that this method of operation, wherein the aponeurotic and muscular layers composing the abdominal wall are successively and cautiously divided is better than the plan which has been proposed of plunging a bistoury directly into the abscess and then enlarging the wound as the knife is withdrawn. Such a course can only be proper when fluctuation is present, and the matter lies near the surface. Nevertheless I would avoid the extensive incisions which are sometimes recommended, and which have been known to be followed by hernial protrusion. An external incision of two inches will afford ample space ; and the wound should grow narrower as it increases in depth, while the direct opening into the abscess need not be larger than will readily admit the forefinger. This I think it desirable to insert, in order to ascertain the extent of the cavity, and to detect, if possible, the presence of foreign bodies or fæcal concretions. When these are found, they should always be removed ; otherwise they may cause future trouble. In a case which I treated by incision several years ago, the patient did well after the operation, and returned to his work. About a month later, however, suppuration recurred, the wounds re-opened, and allowed the escape of a small fæcal concretion, the discharge of which was followed by a permanent cure.

Concretions were found in four out of the eleven cases treated by incision. One of them was large, and resembled in size and shape a date pit; the others were small, and, like the large one, consisted of inspissated fæcal matter, arranged in the form of concentric laminæ. One of them contained a few raspberry seeds, and in one instance, eight concretions, evidently formed in the appendix, were obtained from a single patient. Probably similar formations existed in other cases, but were overlooked and thrown away with the discharges. The contents of the abscesses were always exceedingly fetid, had a fæcal odor, and were more or less gaseous, rendering it probable that the intestine had been perforated. It is not a little remarkable that these perforations of the intestine, evident in some instances, and probable in all, invariably closed, for in no case did a fistulous track remain as a sequel of the operation. In the earlier cases the abscesses were kept clean by daily injections of tepid water; in the later ones a drainage tube was inserted and the injection made antiseptic by the admixture of carbolic acid.

My notes contain several cases in which perityphlitis occurred more than once in the same patient. In one of these the second attack, which terminated by resolution, took place thirteen months after a successful operation for abscess. No concretion or foreign body was found at the time when the abscess was opened, and it is possible that the retention of some such extraneous substance may have caused the subsequent trouble. This supposition is favored by the history of two other cases given in the table, in one of which there had occurred within a period of two years no fewer than three attacks of perityphlitis, the last one only ending in abscess. In the other patient an abscess formed two and a half years after a sharp attack of perityphlitis, terminating in resolution. In both, fæcal concretions were discharged from the abscess; and it is fair to presume that these had been the cause of the previous attacks. Such cases teach us that we should be guarded when giving a prognosis respecting the liability to a recurrence of the disease, after recovery has taken place by resolution. If the appendix contains a concretion, this will probably excite renewed irritation until the offending substance is discharged by suppuration.

In opening perityphlitic abscesses, in which fluctuation could not be detected, I have always proceeded with caution, for fear of wounding the peritoneum or the intestine; but I am doubtful whether such caution is absolutely necessary. Whether in consequence of the fact that these abscesses contain gas, or because they are adjacent to the intestine, they often yield a tympanitic sound on percussion, giving one the impression that the intestine is close at hand. But I have never observed this to be the case on opening the abscess, which is frequently so large that the fore-finger inserted into it can barely touch the cæcum, displaced from its normal situation toward the median line.

It is generally assumed that when abscess results from perforation of the appendix, the matter is contained in the peritoneal sac, a portion of which is shut off from the rest by adhesions between the intestines, the parietal peritoneum, or the omentum. That the abscess may be thus constituted I am well aware; but I believe that such a mode of origin is quite exceptional, and that when, in consequence of intestinal perforation fæcal matters escape directly into the peritoneal cavity, the result is almost invariably a diffused septic inflammation of the peritoneum, ending in speedy death. Pathological anatomy has shown the possibility of another mode of abscess-formation, which I believe to be far more common. The vermiform appendix, before becoming perforated, may contract adhesions to the peritoneum lining the iliac fossa, on which it usually rests. Consequently, when the coats of the appendix have been destroyed, the ulceration extends through the apposed layer of peritoneum in such a manner, that the fæcal matters, instead of entering the serous sac, gradually pass into the loose connective tissue which lies outside the peritoneum, and there set up suppurative inflammation. The pus, as it accumulates, may burrow behind the cæcum and ascending colon; or it may descend behind the peritoneum into the pelvis; or, as most often happens, it may occupy more or less completely the iliac fossa. In the latter case, the serous membrane, which is here very loosely adherent to the iliac fascia, will be detached and deflected toward the median line, carrying with it, in the same direction, the cæcum and the small intestine. Here there will be little danger of wounding the peritoneum while opening the abscess, provided the operator avoids the upper and inner margins of the tumor, where the serous membrane forming the boundary of the abscess is reflected upon the anterior abdominal wall. Of course, in the event of an erroneous diagnosis, grave accidents might occur, for an incision which, in the case of abscess, would simply enter the suppurating cavity, might otherwise penetrate the peritoneal sac and perhaps also involve the intestine. The aspirator offers, as has been stated, the best safeguard against such a blunder, and should invariably be employed in doubtful cases.

I have but a few words to add concerning the fourth and last group of cases, those in which the abscess terminated fatally, without discharging its contents either internally or externally. This group comprises only two cases, both of which possess features of interest. One of them I saw six years ago, in consultation with Dr. Smith Ely, of Newburgh. The patient was a gentleman forty-eight years of age, who after having suffered for some time with the symptoms of inflammation in the region of the cæcum, was seized with general peritonitis. At the time when he came under my observation, I found the abdomen greatly distended, but could discover no tumor in the iliac fossa or in the rectum. He declined to submit to the usual exploratory operation, but allowed me to cut through the skin and the thick subcutaneous fat, and to insert the needle of a hypodermic syringe into the deeper tissues. This was done with a negative result. Death occurred from peritonitis, and a post-mortem examination revealed an extensive abscess behind the cæcum and ascending colon, reaching as high as the under surface of the liver, and communicating with the intestine through an ulcerated opening in the posterior wall of the cæcum. The abscess was filled with pus and blood, and did not open into the peritoneum. The vermiform appendix was intact. This case, as well as the one I am about to relate, shows that a perityphlitic abscess may be situated altogether behind the colon, and suggests the propriety of inserting an aspirating needle through the posterior wall of the abdomen, when the symptoms of perityphlitis are present without the development of the usual iliac swelling. Should matter be found, it could then be evacuated by an incision like that usually made in the operation of colotomy.

The second patient, a gentleman forty years of age, I saw in consultation with Drs. Rodenstein and Otis. The history of the disease pointed clearly to perityphlitis, but there was no tumor. Digital exploration of the rectum failed to discover any swelling, but detected slight tenderness high up on the right side. On the fourth day the patient became somewhat delirious, and on the sixth day he had a convulsion. From that time until his death, which took place on the sixteenth day, the symptoms were those of cerebral inflammation, the patient dying comatose. A post-mortem examination discovered the changes in the brain characteristic of purulent meningitis; and the disease in this case seemed to be pyæmic, for, on opening the abdomen, an abscess containing eight ounces of fetid pus was found situated in the lumbar region, behind the cæcum and ascending colon. The abscess communicated with the vermiform appendix, which was the seat of a double perforation. No tumor existed in the iliac fossa. There were no evidences of peritonitis except the presence of some adhesions connected with the appendix.

The early supervention, in this case, of acute cerebral inflammation would have prevented the success of any surgical operation, even had the situation of the abscess been known during life. But the case is instructive as showing that a perforating ulcer of the appendix, like a similar ulcer in the back of the cæcum, may give rise to an abscess in the lumbar region that cannot be discovered by the ordinary method of examination.—*Annals of the Anat. & Surg. Soc'y*, July, 1880.

